# Preparation of amorphous silicon-doped Y_2_O_3_ aerogel enabling nonlinear optical features for ultrafast photonics

**DOI:** 10.1515/nanoph-2023-0894

**Published:** 2024-03-13

**Authors:** Qingxi Zhao, Hongwei Chu, Zhongben Pan, Benxue Liu, Han Pan, Shengzhi Zhao, Dechun Li

**Affiliations:** School of Information Science and Engineering, Key Laboratory of Laser and Infrared System of Ministry of Education, 12589 Shandong University, Qingdao 266237, China; State Key Laboratory of Crystal Materials, 12589 Institute of Crystal Materials, Shandong University, Jinan 250100, China

**Keywords:** Y_2_O_3_ aerogel, nonlinear optical properties, saturable absorber, mode-locked lasers, fiber lasers

## Abstract

Amorphous aerogels with the microscopic nanoscale three-dimensional meshes provide superb platforms for investigating unique physicochemical properties. In order to enhance the physical, thermal and mechanical performances, one efficient and common approach is integrating diverse functional materials. Herein, we report a simple strategy to fabricate the amorphous silicon doped Y_2_O_3_ aerogels with the post-gelation method under the N_2_/EtOH supercritical atmosphere. The impact of Si concentration on the nonlinear optical properties is investigated for the first time. The maximum modulation depth is 1.65 % with a saturation intensity of 0.78 MW cm^−2^ with the 1-ps laser excitation at 1590 nm. Finally, we incorporated the silicon-doped Y_2_O_3_ aerogel based saturable absorber (SA) into an erbium-doped fiber laser (EDFL) and achieved various mode-locking operations at different wavelengths in the super C band, in terms of the conventional soliton, harmonic soliton molecules pulses, and dual-wavelength soliton mode-locking. Overall, this work confirms that amorphous silicon-doped Y_2_O_3_ aerogels are good nonlinear optical materials and pave a way for the ultrafast photonic and nonlinear optical applications with amorphous materials in near future.

## 1 Introduction

Aerogels possess a three-dimensional mesoporous network with ultralow thermal conductivity and high specific surface area [1], [2]. Owing to the unique structure, aerogels exhibit numerous properties such as high specific surface area, large porosity, ultra-low thermal conductivity, ultra-low dielectric constant, the tailored density, and the manipulated refractive index [3], [4], [5]. Therefore, aerogel materials have garnered widespread attention and been successfully applied in fields such as thermal insulation [6], biomedicine [7], impurity adsorption [8], [9], and catalyst [10]. Moreover, after the special manufacturing and processing, favorable properties of aerogels can even be efficiently enhanced [11].

In fact, SiO_2_ aerogels are the earliest invented [12] and commercialized materials. Although the strength-weight ratio in the aerogels is extremely high [13], SiO_2_ aerogels are normally fragile [14]. With the exhaustive investigation and methodology evolution, versatile metal oxide aerogels [15]–[19] have been successively developed including ZrO_2_, Al_2_O_3_, and TiO_2_. The advent of the metal oxide aerogels instills surprised electronic, mechanical, and surface/interface properties. However, the metal oxide aerogels suffer the instability especially at the higher temperatures [20], [21]. In recent years, Y_2_O_3_ has been poured attention in because of the high melting temperature and excellent physicochemical stability. In addition, as a third-order nonlinear optical response, the saturable absorption in Y_2_O_3_ can be applied in the fiber lasers to realize the Q-switching and mode-locking operations [22], [23], [24]. Therefore, researchers pay great attention to Y_2_O_3_ materials. In recent years, the field of ultrafast photonics has been rapidly developed, and various achievements have emerged in an endless stream [25]–[29]. Among them, passive mode-locking fiber lasers have been widely studied because of their self-started operation, excellent beam quality and compact structure [30], [31], [32]. Previous studies have also shown that Y_2_O_3_ is a promising material in the field of nonlinear optics.

However, for the metal oxide aerogels fabrication, owing to the hydrolysis and the condensation of the precursor, the aerogels are typically amorphous. The amorphous materials normally exhibit the distinct features owing the symmetry breaking and disorder. Compared with crystalline materials, amorphous materials do not have long-range ordered periodicity, which is conducive to the generation of localized electrons/holes in the band gap [33], [34], [35]. The structural disorder of amorphous materials can promote charge capture in the materials through the relaxation of the local atomic environment. In fact, the intrinsic electron and hole capture in amorphous oxide semiconductors have been demonstrated by theoretical calculations and experiments [36], [37], [38]. The charge-trapping effect of amorphous materials is much greater than that of the same kind of crystalline materials, and the quantity of electric charges trapped increases with the increase of the disorder, which leads to electrons-holes separation, thus enhancing the nonlinear optical properties of amorphous oxide semi-conductors [39], [40]. Thus far, many amorphous molecular materials have been paid attention to and studied, and have been applied for the supercontinuum generation [41]. However, as far as we know, there are few reports about the application of amorphous materials in ultrafast lasers, which has aroused our concern.

In this paper, we fabricated the amorphous Si-doped Y_2_O_3_ aerogels using the post-gelation method under the supercritical atmosphere. Under the different conditions, we synthesized the Si-doped Y_2_O_3_ aerogels with different Si concentration. The impact of the Si concentration on the nonlinear optical properties was comprehensively studied. The highest modulation depth was 1.65 % with a saturation intensity of 0.78 MW/cm^2^ with the 1-ps 1590-nm laser pulse excitation, demonstrating the favorable nonlinear optical properties of the Y_2_O_3_-based saturable absorber. Finally, we incorporated the Si-doped Y_2_O_3_ aerogel based saturable absorber (SA) into an erbium-doped fiber laser (EDFL). By adjusting the evanescent light field interactions with the amorphous aerogel, various soliton mode-locking operations in the super C band were achieved, producing the conventional soliton pulses, harmonic soliton molecules pulses, and dual-wavelength soliton pulses. Our work indicates that amorphous Y_2_O_3_ aerogel is a great potential nonlinear optical material and will expand the future applications of the amorphous materials in the ultrafast photonics.

## 2 Results and discussion

### 2.1 Characterization of amorphous silicon-doped Y_2_O_3_ aerogel

**Figure 1:**
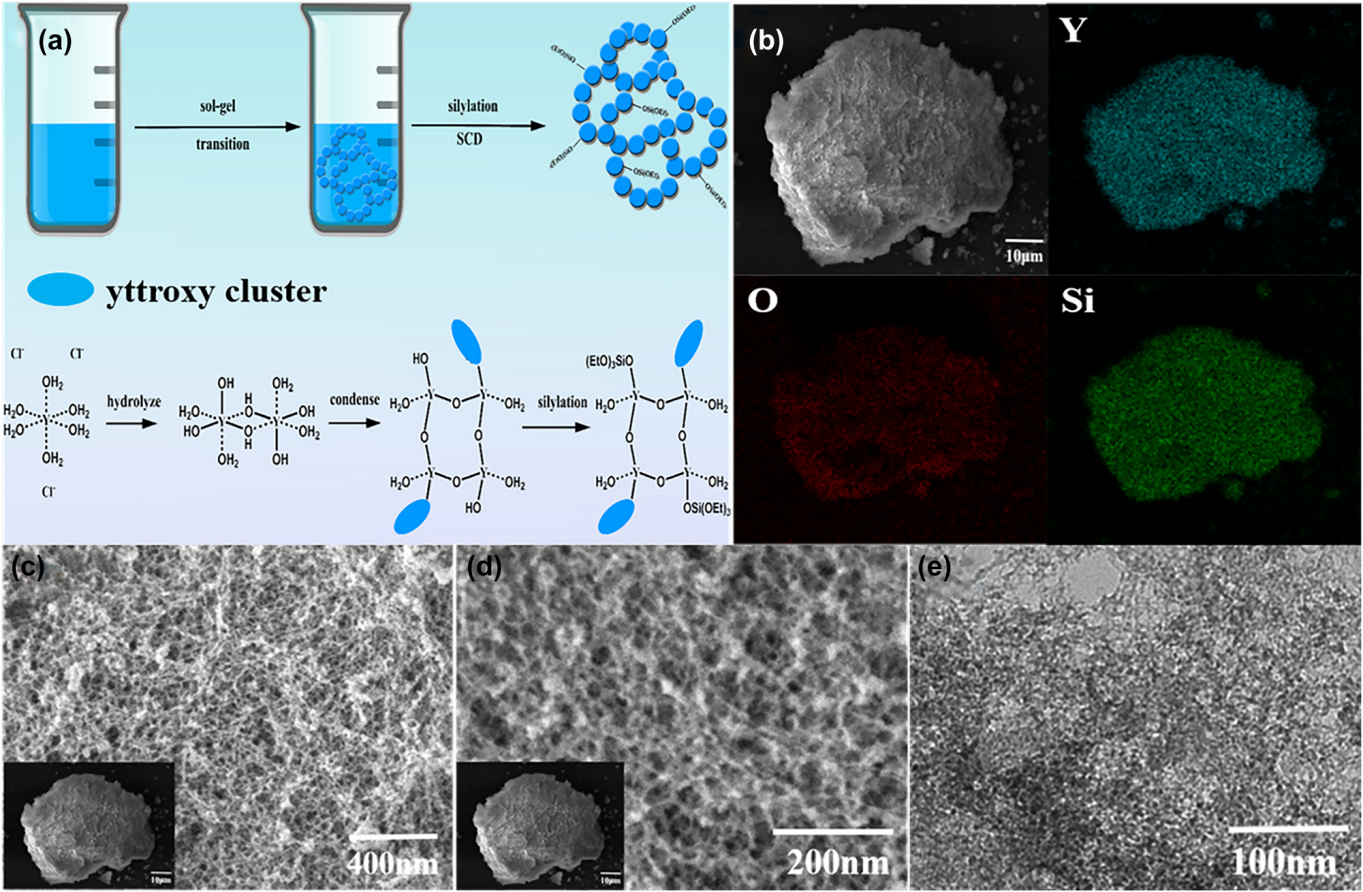
Fabrication and Characterization. (a) Graphical illustration of the formation of Y_2_O_3_-3 gel networks. (b) The energy-dispersive X-ray spectroscopy (EDS) elemental mappings of amorphous Y_2_O_3_ aerogel. (c) And (d) SEM images at different scales. (e) TEM image.

Based on previous studies [42], [43], [44], it has been established that yttrium ions exhibit a coordination number of eight with water molecules in aqueous solutions, owing to their heightened electrophilicity. In the current investigation, a precursor of yttrium chloride hexahydrate was dissolved in absolute ethanol; the yttrium ions were anticipated to undergo hydration, forming connections with six water molecules, which is the maximum possible coordination due to an insufficient quantity of available water molecules. In this context, it was postulated that the yttrium ions, coordinated with six water molecules, would undergo hydrolysis, releasing hydrogen ions subsequent to the introduction of a propylene oxide (PO) gel initiator. The hydroxyl yttrium species would then proceed to polycondense, leading to the formation of bi-continuously connected Y_2_O_3_ gel networks through a concurrent phase separation process. The entire detailed process is shown in Figure 1(a). In order to introducing the Si ions, tetraethyl orthosilicate (TEOS)/ethanol (EtOH) solutions with different volume ratios were utilized. In our case, the wet gel which was not treated with TEOS/EtOH solution was labeled as Y_2_O_3_-1 aerogel, actually consisting of no Si concentration at all. The wet gels treated in 25 vol% and 50 vol% TEOS/EtOH solutions for one day were denoted as Y_2_O_3_-2 and Y_2_O_3_-3 aerogels, respectively. Indeed, after the TEOS/EtOH treatment, the aerogel was of more stable structural integrity. The typical microscopic structure and morphology of the prepared amorphous Y_2_O_3_-3 aerogel were performed with the scanning electron microscopy (SEM) and the transmission electron microscopy (TEM). The corresponding element mapping images of amorphous Y_2_O_3_ aerogel are shown in Figure 1(b), which clearly show the distribution of elements Y, O, and Si. The typical SEM images with different resolution are displayed in Figure 1(c) and (d). Obviously, SEM images uncovered the irregular pores in the prepared Y_2_O_3_ aerogel with a size of ∼30 nm. The inset of Figure 1(c) and (d) shows the overall morphology of the sample at a scale of 10 μm. Figure 1(e) shows a typical TEM image captured at a scale of 100 nm, further revealing the porous structure of the Y_2_O_3_ aerogel material. The SEM and TEM images of Y_2_O_3_-1 and Y_2_O_3_-2 can be seen in Figures S1 and S2.

**Figure 2:**
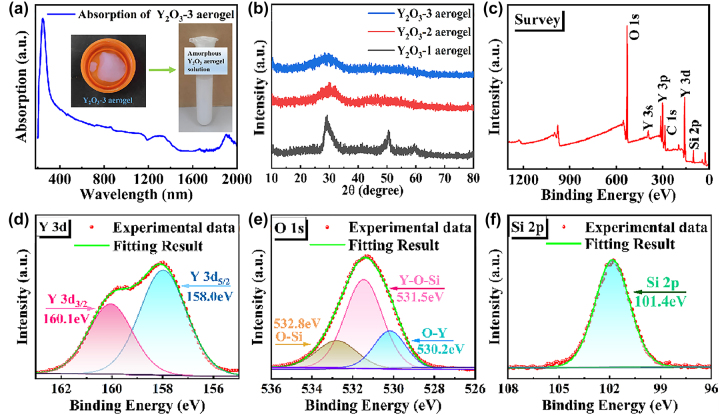
Characterization. (a) UV–VIS–IR absorption spectra of Y_2_O_3_ aerogel. (b) XRD pattern. (c–f) XPS of amorphous Y_2_O_3_ aerogel.

To investigate the optical absorption properties of the prepared amorphous Y_2_O_3_ aerogel, the sample was ground into powder, then dissolved into anhydrous ethanol solution for ultrasonic and centrifugal treatment, and the supernatant was taken for use. The optical absorption characteristics of the material were measured using a UV–VIS–IR spectrophotometer. Clearly, as shown in Figure 2(a), the amorphous Y_2_O_3_ material exhibits broad absorption characteristics in the near-infrared range, suggesting the great potential for various photonics applications. Figure 2(b) represents the X-ray diffraction (XRD) analysis of the Y_2_O_3_ aerogels. It can be seen that with the incorporation of Si, the crystallinity of Y_2_O_3_ aerogels continues to deteriorate, and the reason is analyzed as follows: The Si^4+^ ions have a significantly smaller ionic radius (*R*_Si4+_ = 0.04 nm) compared to the Y^3+^ ions (*R*_Y3+_ = 0.09 nm). As a result Si doping causes stress in the Y_2_O_3_ lattice, degrading its crystal structure [45]. To confirm the chemical bonding in the amorphous silicon-doped Y_2_O_3_ aerogel, the X-ray photoelectron spectroscopy (XPS) analysis was carried out. Figure 2(c) shows the distinct evidence of the existence of Y 3d, O 1s, and Si 2p peaks. For the Y 3d binding spectrum, there are two prominent peaks at 158.0 and 160.1 eV in Figure 2(d), which can be assigned to the divisive Y 3d_5/2_ and Y 3d_3/2_ states [46]. Displayed in Figure 2(e), the XPS spectra of O can be deconvoluted into three peaks at 530.2, 531.5, and 532.8 eV, respectively. The binding energy of 530.2 eV came from the O–Y bond. While the other peaks at 531.5 and 532.8 eV indicated the presence of the Y–O–Si and O–Si bonds, suggesting the incorporation of Si during the material synthesis process. Furthermore, the only peak of Si 2p spectrum in Figure 2(f) is observed at 101.4 eV, which can be attributed to the inhomogeneous broadening in the non-crystalline aerogel.

Additionally, in order to study the effect of silicon concentration on the optical properties of Y_2_O_3_ aerogels, X-ray diffraction (XRD) patterns, UV–VIS–IR absorption spectra and the Fourier-transformed infrared (FT-IR) spectra were used to analysis the Y_2_O_3_ aerogels, as shown in Figure S3. Clearly, with the Si concentration increase, the aerogel was getting more and more amorphous, while the absorption peak attributed to the Si–O bond was stronger, indicating that the Si incorporation degraded the crystal structure.

### 2.2 Nonlinear optical properties in Y_2_O_3_ aerogels

**Figure 3:**
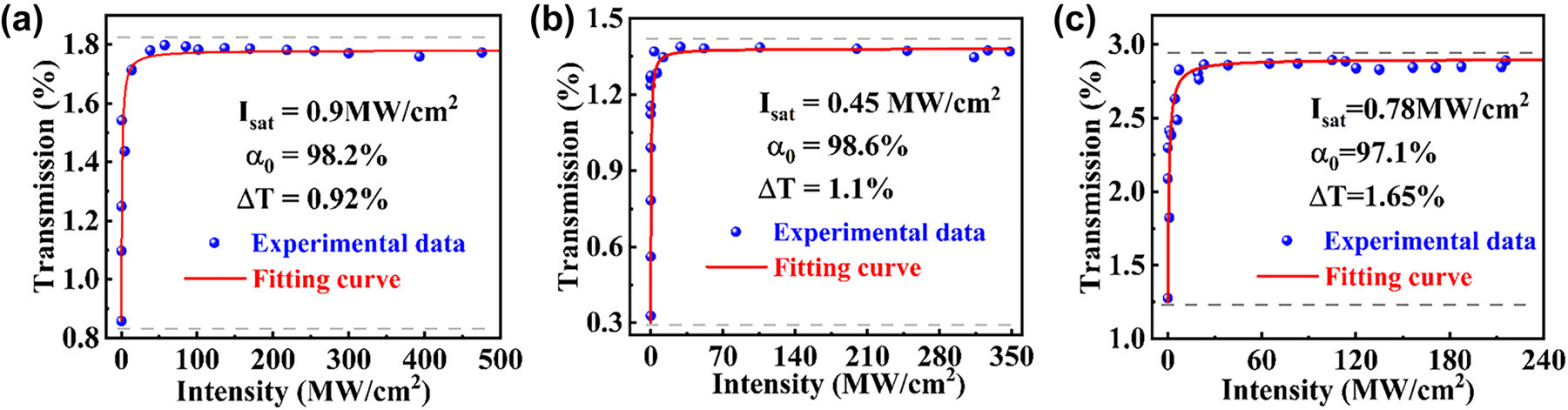
Nonlinear transmission curve of Y_2_O_3_ aerogel with different silicon concentration. (a) Y_2_O_3_-1 aerogel. (b) Y_2_O_3_-2 aerogel. (c) Y_2_O_3_-3 aerogel.

The nonlinear optical absorption measurement was carried out with an in-line twin-detector system excited with the picosecond 1590-nm fiber laser, as shown in the Supplementary Material. Obviously, the nonlinear transmission curve of the Y_2_O_3_ aerogels can be fitted by [47]:

(1) $$T=1-\frac{\Delta T}{1+\frac{I}{I_{\text{sat}}}}-\alpha_{0},$$

here, *T*, Δ*T*, *I*, *I*_sat_, and *α*_0_ denote the transmission, modulation depth, on-axis pump intensity, the saturation intensity and the unsaturation loss of the prepared samples. Obviously, all samples in Figure 3 possess the typical saturable absorption effect at 1590 nm. The Y_2_O_3_-3 aerogel exhibits the largest modulation depth of 1.65 % with a relative low saturation intensity of 0.78 MW/cm^2^. The saturable absorption properties enabled the mode-locking performance to generate the ultrafast optical pulses.

### 2.3 Mode-locking EDF laser near 1530 nm

The nonlinear optical (NLO) responses of Y_2_O_3_-based SAs show that they have admirable saturable absorption characteristics. Therefore, to compare their potential in ultrafast photonics applications, they were coupled into the ring Er-doped fiber laser cavity operating in the super C band. It is worth noting that hereby we only demonstrated the mode-locking operation with the Y_2_O_3_-3 aerogel SA, owing to the large modulation depth. The detailed experimental results from the passively mode-locked EDFLs with Y_2_O_3_-1 and Y_2_O_3_-2 aerogels SAs are attached in the Supplementary Material.

**Figure 4:**
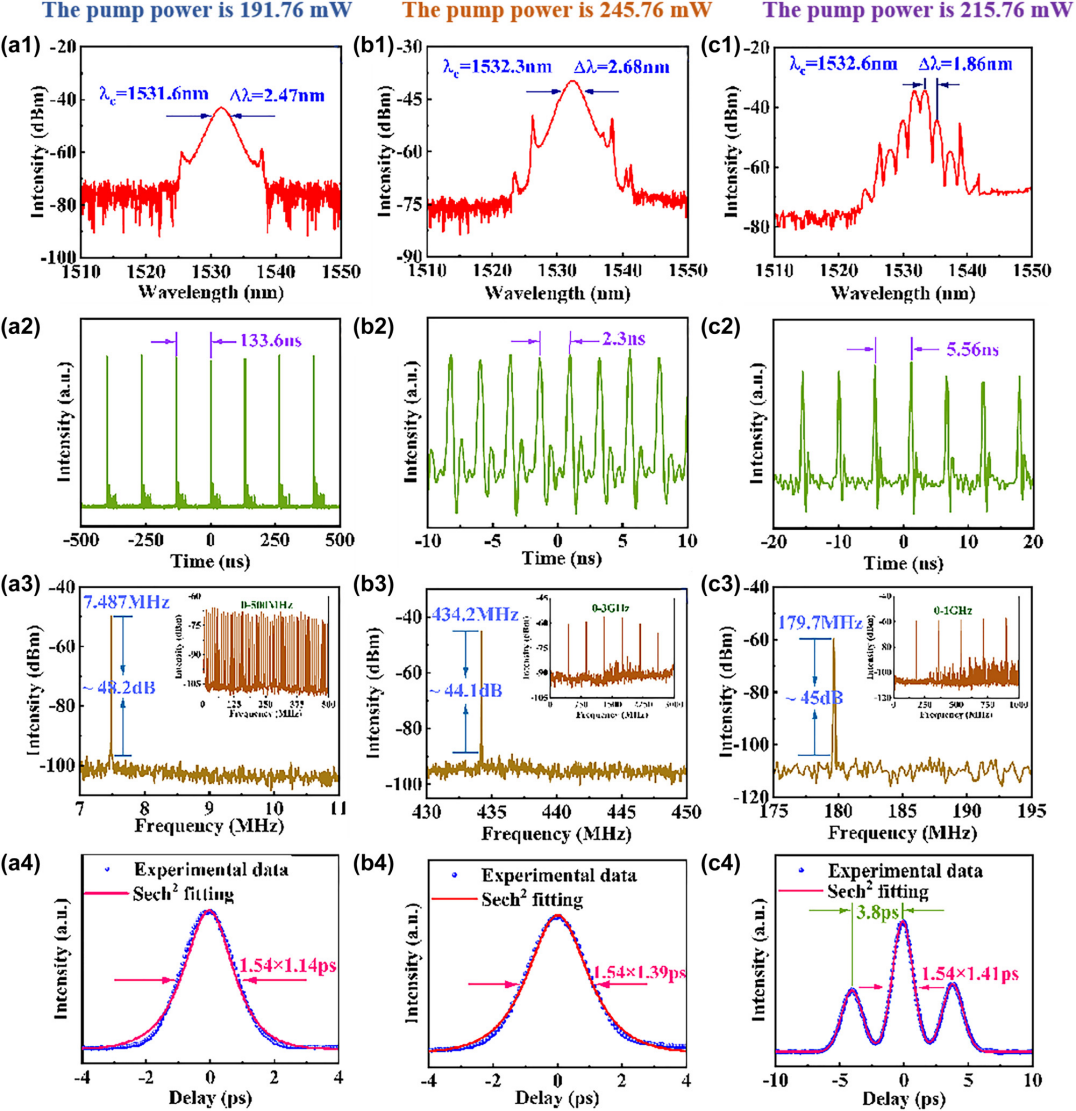
Mode-locking EDF laser near 1530 nm. (a1–a4) Conventional soliton operation; (a1) optical spectrum, (a2) pulse trains, (a3) RF spectrum, inset: the broad-range RF spectrum, (a4) autocorrelation trace. (b1–b4) Harmonic conventional soliton operation; (b1) optical spectrum, (b2) pulse trains, (b3) RF spectrum, inset: the broad-range RF spectrum, (b4) autocorrelation trace. (c1–c4) Harmonic soliton molecules operation; (c1) optical spectrum, (c2) pulse trains, (c3) RF spectrum, inset: the broad-range RF spectrum, (c4) autocorrelation trace.

By tuning the polarization state and pump power, the mode-locking pulses of the EDFL near 1530 nm are realized, as shown in Figure 4. Figure 4(a) shows that the mode-locked laser runs in the conventional soliton mode-locking operation when the pump power is 191.76 mW. The spectral diagram in Figure 4(a1) shows clear and symmetrical Kelly sidebands. The central spectral wavelength was 1531.6 nm with a 3-dB bandwidth (Full width at half-maximum, FWHM) of 2.47 nm. In fact, the Kelly sidebands are resulted from the constructive interference of the pulse and dispersive wave in the laser resonator [48]. The pulse train in Figure 4(a2) shows the stable time interval of 133.6 ns between the adjacent pulses, coinciding well with the cavity length of 27.31 m. Figure 4(a3) shows the radio frequency (RF) spectrum with a central frequency of 7.487 MHz and a signal-to-noise ratio (SNR) of about 48.2 dB, indicating a stable mode-locking operation. The stability of the mode-locking state was confirmed by the RF spectrum in the 500-MHz range, as shown in the inset of Figure 4(a3). The autocorrelation trajectory in Figure 4(a4) reveals a FWHM of 1.76 ps, corresponding to a pulse width of 1.14 ps with the squared hyperbolic secant fitting.

When the pump power was 245.76 mW, harmonic conventional soliton mode-locking pulses were obtained, as shown in Figure 4(b). Figure 4(b1) shows the spectrum of the harmonic conventional soliton mode-locking operation. The central wavelength was 1532.3 nm, and the 3-dB spectral bandwidth was 2.68 nm with clear and symmetric Kelly sidebands. Figure 4(b2) shows the pulse sequence displayed on the oscilloscope, with adjacent pulses spaced by 2.3 ns. Figure 4(b3) shows the RF spectrum with the SNR of about 44.1 dB, a repetition rate of 434.2 MHz, corresponding to the 58th harmonic mode-locking operation. The stability of the mode-locking state was confirmed by the RF spectrum in the 3-GHz range, as shown in the inset of Figure 4(b3). The autocorrelation trace in Figure 4(b4) possesses a FWHM of 2.14 ps, with a corresponding pulse width of 1.39 ps with the squared hyperbolic secant fitting. The reasons for the formation of harmonic mode-locking operation are explained as follows. For passive mode-locking fiber lasers, with an increase in the pump power, the energy of a single soliton pulse exceeded its maximum saturable energy. Due to the peak power limitation effect and the soliton area theorem, a single soliton pulse was split into multiple pulses to form an unstable multiple-pulse state. In the case of the gain, loss, nonlinearity, and dispersion interaction of the ring cavity, these multiple pulses can equalize via a self-arrangement process and finally form an orderly distributed pulse sequence with uniform spacing, which is referred to as harmonic mode-locking [49], [50].

After obtaining the output of conventional soliton pulses, the pump power was adjusted to 215.76 mW to achieve the output of harmonic soliton molecules pulses, as shown in Figure 4(c). The spectral diagram reveals a central wavelength of 1532.6 nm, and the spectrum exhibits modulation with the modulation period of 1.86 nm, as shown in Figure 4(c1). The pulse sequence diagram shown by the oscilloscope in Figure 4(c2) has an interval of 5.56 ns between the neighbored pulses. In Figure 4(c3), the SNR is about 45 dB, and the repetition frequency is 179.7 MHz, corresponding to the 24th harmonic mode-locking operation. The inset of Figure 4(c3) shows the RF spectrum in the 1-GHz range. The autocorrelation trace with the multi-peak fitting curve is shown in Figure 4(c4), in which the FWHM of the highest peak is 2.17 ps, corresponding to the pulse duration of 1.41 ps and the peak interval is 3.8 ps. The time interval Δ*t* and modulation period Δ*λ* are related as follow [51]:

(2) $$\Delta t=\frac{\lambda_{c}^{2}}{c\cdot \Delta \lambda }$$

The time interval was calculated as 4.2 ps, almost coincident with the experimental data. The formation mechanism of the harmonic soliton molecules pulses can be explained as follows. For the fundamental frequency mode-locked single soliton pulse, the peak power of the optical pulse was clamped due to the quantization effect of the soliton energy attributed to the soliton area mechanism and limited gain bandwidth of the active optical fiber. However, with the increase of pump power, owing to the accumulated nonlinear effect and peak power limiting effect of the soliton, a single soliton pulse splits into multiple pulses with low peak energy. The pulses interact with each other through repulsive and attractive forces. Finally, when the repulsive and attractive forces got a dynamical equilibrium, stable bound solitons are generated [52]. Moreover, under the combined action of cavity gain, loss, dispersion, and cumulative nonlinearity, multiple soliton molecules pulses were automatically arranged to exhibit a uniform amplitude and equal time interval within one round trip. Consequently, the repetition rate of the output pulse was amplified to an integral multiple of the fundamental repetition rate, thus leading to the generation of harmonic soliton molecules pulses [49]. In general, there are both short distance and long distance interactions in the formation process of harmonic soliton molecules mode-locking operation: the former leads to the formation of bound state solitons, and the latter rearranges and distributes the bound state solitons uniformly in the resonator, and finally makes the laser work in the harmonic solitons molecules mode-locking operation [53].

### 2.4 Synchronized dual-wavelength mode-locking near 1530 and 1560 nm

**Figure 5:**
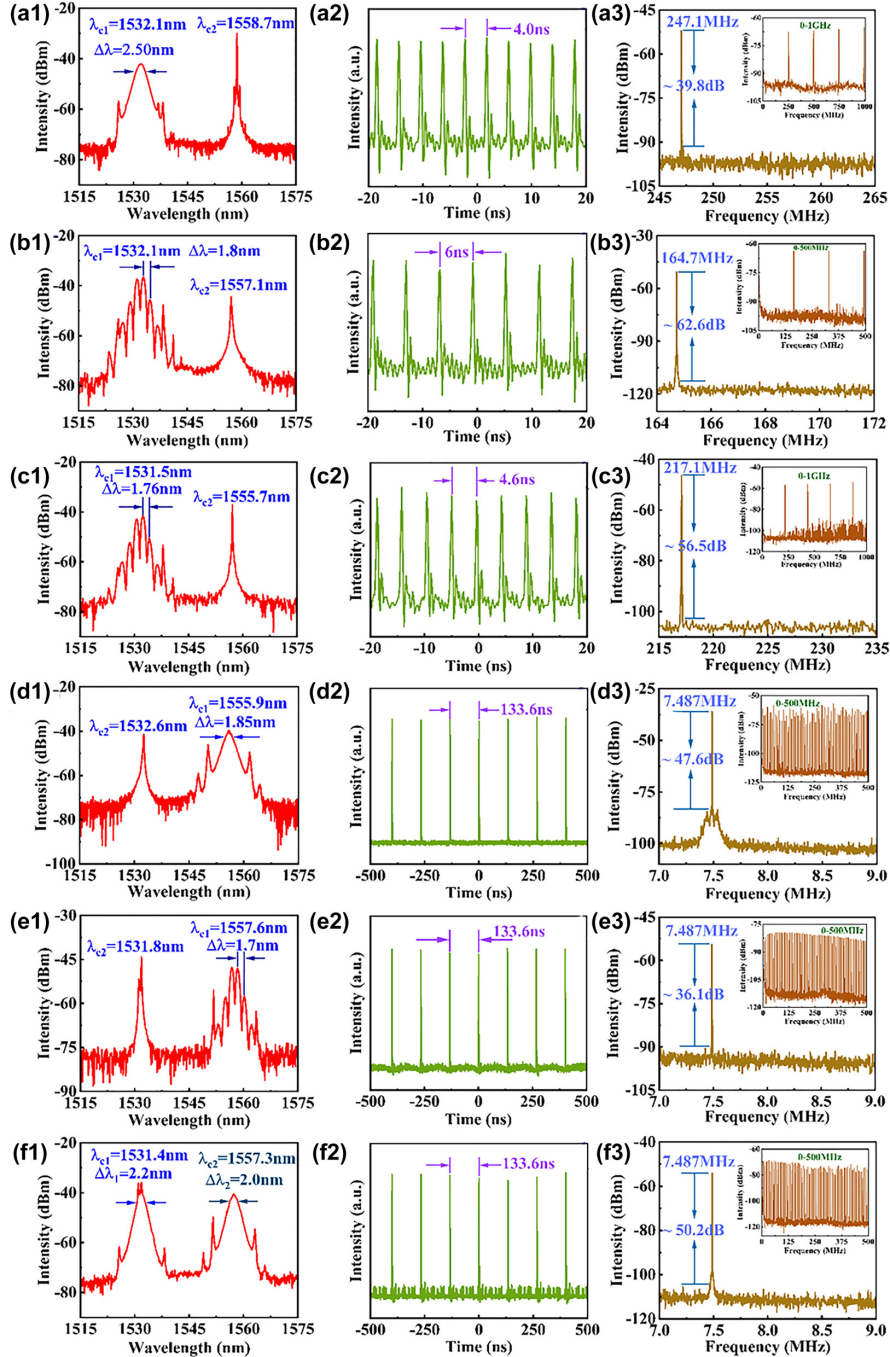
Synchronized dual-wavelength mode-locking near 1530 and 1560 nm. (a1–f1) Optical spectra. (a2–f2) Pulse sequence diagrams. (a3–f3) RF spectra, inset: the broad-range RF spectra.

In addition, synchronized dual-wavelength mode-locking near 1530 and 1560 nm are obtained under different pump power and polarization states, as shown in Figure 5. When the pump power was adjusted to 227.76 mW, the harmonic conventional soliton mode-locked EDFL coexisting with the continuous wave can be achieved after fine tune of the polarization controller. The spectrum of the harmonic conventional soliton mode-locking operation is illustrated in Figure 5(a1), showcasing the continuous wave wavelength at 1558.7 nm and the pulsed wave wavelength at 1532.1 nm, with a 3-dB bandwidth of 2.5 nm. The distinct Kelly sidebands, a typical characteristic of soliton operation, are clearly observed in the Figure 5(a1). Figure 5(a2) displays the pulse sequence obtained from the oscilloscope, with a neighboring pulse interval of 4.0 ns. Figure 5(a3) shows the RF spectrum, with the repetition frequency of 247.1 MHz, corresponding to the 33rd harmonic mode-locking operation, and the SNR of approximately 39.8 dB. The inset of Figure 5(a3) shows the RF spectrum in the 1-GHz range, showing the stability of the mode-locking operation.

When the pump power was adjusted to 203.76 mW, the output of 22nd harmonic soliton molecules is obtained by tuning the polarization state, as shown in Figure 5(b1). The central wavelength of the soliton molecules was 1532.1 nm and the modulation period was 1.8 nm. There was an obvious continuous wave component in the spectrum with a central wavelength of 1557.1 nm. The adjacent pulse interval was 6 ns, corresponding to repetition rate of 164.7 MHz, and the SNR is as high as 62.6 dB. The inset of Figure 5(b3) shows the RF spectrum in the 500-MHz range. Increasing the pump power to 227.76 mW, the output of the 29th harmonic soliton molecules can be obtained, as shown in Figure 5(c). The central wavelength of the soliton molecules was 1531.5 nm and the modulation period was 1.76 nm. There was also an obvious continuous wave component with a central wavelength of 1555.7 nm. The SNR is about 56.5 dB, the repetition rate is 217.1 MHz, corresponding to an adjacent pulse interval of 4.6 ns. The inset of Figure 5(c3) shows the RF spectrum in the 1-GHz range, which demonstrates the stable mode-locking operations.

Increasing the pump power to 245.76 mW and adjusting the polarization controller, we were able to achieve conventional soliton mode-locking operation and soliton molecules mode-locking operation near 1560 nm. The conventional soliton mode-locked laser operated at 1555.9 nm with a FWHM of 1.85 nm was realized, as depicted in Figure 5(d1). The continuous wave component operated at 1532.6 nm. The pulse sequence displayed by the oscilloscope, as shown in Figure 5(d2), has a pulse interval of 133.6 ns, which matches the cavity length. In Figure 5(d3), the fundamental repetition frequency is 7.487 MHz, which is consistent with the conventional soliton mode-locking operation at the wavelength of 1531.6 nm with an SNR of about 47.6 dB. The inset shows the mode-locking pulse RF spectrum within a span of 500 MHz. Figure 5(e) shows the soliton molecules mode-locking operation near 1560 nm. In Figure 5(e1), we find that the spectrum exhibits modulation with a modulation period of 1.7 nm and the central wavelength of 1557.6 nm and an obvious continuous-wave spectral component at 1531.8 nm. Figure 5(e2) shows the pulse interval is 133.6 ns. The SNR in Figure 5(e3) is about 36.1 dB, the repetition frequency is 7.487 MHz. The RF spectrum in the range from 0–500 MHz was displayed in inset of Figure 5(e3).

Finally, we achieve the synchronized dual-wavelength conventional soliton mode-locking operation near 1530 and 1560 nm. After maintaining the pump power at 245.76 mW, the polarization state was carefully tuned to achieve the dual-wavelength conventional soliton mode-locking operation. The broad gain bandwidth of Er-doped fiber enabled the laser to produce synchronized conventional soliton pulses at two different central wavelengths [54]. The dual-wavelength mode-locking spectrum, shown in Figure 5(f1), displays two separated spectral peaks at 1531.4 nm and 1557.3 nm, with 3-dB bandwidths of 2.2 nm and 2.0 nm, respectively. It can be observed that continuous wave is present in the spectrum. Figure 5(f2) displays the pulse sequence at this point, with a pulse interval of 133.6 ns, agreeing with the conventional soliton mode-locking operation when the pump power was 191.76 mW. In Figure 5(f3), the SNR is about 50.2 dB. The fundamental repetition frequency is 7.487 MHz, and the inset displays the RF spectrum in the range from 0–500 MHz, indicating the stable mode-locking operation. Owing to the wide gain band of EDFL and the high nonlinearity of SA, the laser has an approximate gain level near 1531.4 nm and 1557.3 nm, and can realize the synchronized dual-wavelength pulse output under specific pump power and polarization state. Besides, the continuous light at the top of the spectrum is the result of the interaction between the birefringence effect of the fiber and the nonlinear optical effect of SA [55].

## 3 Experimental section

### 3.1 Synthetic methods

Yttrium chloride hexahydrate (YCl_3_·6H_2_O, Aladdin, 99.5 %) and PO (Aladdin, 99.5 %) were utilized as the primary raw materials in this process. To initiate the synthesis, YCl_3_·6H_2_O was dissolved in ethanol (EtOH, Fuyu, 99.5 %) within a polyethylene mold, and the resulting solution was stirred for 30 min until a clear sol solution was obtained. Following the sol preparation, the solution was then cooled in an ice bath until reaching a temperature of 0 °C. Subsequently, a measured quantity of PO was slowly added drop by drop to the sol. In this case, the YCl_3_·6H_2_O EtOH solution had a molar concentration of 0.5 mol/L. The final sol with the PO addition, the molar ratio of Y^3+^ and PO was set as 1:4. After a 5-min stirring period, the solution was allowed to undergo gelation under standard room temperature conditions (25 °C, 1 bar) and aged for 1 h. During the gelation process, which lasted approximately 10 min, the flowable sol transformed into a gel structure. Two of three wet gels were solidified by immersing in 25 vol% and 50 vol% TEOS/EtOH solution for one day, respectively. To remove any impurities and by-products, all three wet gels were thoroughly washed with EtOH, a process repeated 6 times with 12-h intervals between each washing step. Finally, the wet gel was subjected to supercritical drying at 280 °C under a pressure of 100 bar for 24 h, employing an N_2_/EtOH supercritical atmosphere. This step allowed for the effective removal of the liquid phase, resulting in the formation of the desired Y_2_O_3_ aerogel. The one without treatment in TEOS/EtOH solution, consisting of no silicon, was labeled as Y_2_O_3_-1 aerogel. The aerogels treated in 25 vol% and 50 vol% TEOS/EtOH solutions were denoted as Y_2_O_3_-2 and Y_2_O_3_-3 aerogels, respectively.

### 3.2 Fabrication of silicon-doped Y_2_O_3_ aerogel saturable absorber

Amorphous silicon-doped Y_2_O_3_ aerogel (Y_2_O_3_-3) was ground into the powder and added to anhydrous ethanol to create a suspension, from which the supernatant was collected. Then, the partial coating of a single-mode fiber (SMF-28e, Corning Inc.) was removed using wire strippers. Then, we fixed the ends of the stripped coated part of the fiber and heat the exposed part of the fiber with an alcohol lamp. In the meantime, the fiber was slowly stretched from both ends to create a tapered shape. The minimum waist diameter was 9.6 µm, and the length of the tapered section was 6.0 mm using a microscope. At this time, the insertion loss was measured to be about 97 %. Finally, the Y_2_O_3_ supernatant was dropped onto the tapered fiber, while the CW laser beam at 974 nm propagated in the tapered fiber to promote the optical deposition on the tapered fiber. After deposition, the insertion loss of Y_2_O_3_ – based saturable absorber was measured to be 98 %. The preparation process of the other two Y_2_O_3_-based SAs is the same as described above. In order to ensure the consistency of experimental conditions as much as possible, the insertion loss of the new tapered fibers was kept consistent with that of the tapered fiber mentioned above when we prepared them, and the insertion loss was still consistent after the deposition of the material. The preparation process is detailed in Supplementary Material.

### 3.3 Characterization

The X-ray diffraction patterns were obtained by an X-ray diffactometer (Shimadzu XRD-6100). The X-ray photoelectron spectrum was performed on a XPS system (Thermo fisher scientific K-alpha). The morphologic images were carried out with the scanning electron microscope (FEI Quanta 250 FEG) and the transmission electron microscope (FEI Tecnai G2 F20). The UV–VIS–IR absorption and the Fourier transformed infrared absorption were implemented with a UV-VIS-NIR spectrometry (Shimadzu UV-3600) and FTIR spectrometer (Thermo Fisher Scientific Nicolet iS 5).

### 3.4 In-line twin-detector system

To study the optical properties of the amorphous silicon-doped Y_2_O_3_ aerogel, we characterized nonlinear absorption properties using a home-made twin-balanced-detector system. A passively NPE mode-locked EDFL running at 1590 nm was utilized as the excitation laser with a pulse duration of 1.1 ps. Then in order to ensure the enough output power for the nonlinear optical absorption measurement, an erbium-doped fiber amplifier (EDFA) was applied to further boost the excitation intensity. A variable optical attenuator (VOA) was used to adjust the laser power input into an optical coupler (OC). The output beam from the OC was divided into two sub-beams by a 50:50 tap coupler: One sub-beam was directly detected with a power meter as the reference and the other beam entered an identical power meter after passing through the saturable absorber. The schematic diagram of in-line balanced twin-detector system is provided in Supplementary Material.

### 3.5 Laser configuration

The 974-nm laser diode (LD) emitted the pump beam into the laser cavity via a 980/1550 wavelength division multiplexer (WDM). The gain medium of the cavity was a 1.48-meter-long Erbium-doped fiber (EDF, Fibercore I-25) with a group velocity dispersion (GVD) of 40 ps^2^/km. A polarization-independent isolator (PI–ISO) and polarization controller (PC) were used to maintain unidirectional transmission of the laser beam and adjust the polarization, respectively. The total fiber length of the cavity was 27.31 m, including 25.83 m of single-mode fiber with a GVD of −22.3 ps^2^/km. A 10:90 tap coupler was used to filter out 10 % of the power and the rest of beam was reflected back to the laser resonator. To analyze the output mode-locked pulses, several measurement instruments were used, including an oscilloscope (MDO4104C, Tektronix Inc.), an InGaAs photodetector (3 GHz bandwidth), a spectrum signal analyzer (FPC1000, Rohde&Schwarz Inc.), a spectral analyzer (6375D, Yokogawa Inc.), and an autocorrelator (FR-103XL, Femtochrome Inc.).

## 4 Conclusions

In this paper, amorphous silicon-doped Y_2_O_3_ aerogel was prepared by sol–gel method, and the microstructure, elemental composition and optical absorption characteristics were studied. The introducing Si can efficiently distort the Y_2_O_3_ crystal structure, leading to the amorphous phase. Subsequently, the amorphous silicon-doped Y_2_O_3_ aerogel was optically deposited on a tapered fiber as a saturable absorber for the ultrafast mode-locking operation. The largest modulation depth was 1.65 % with a saturation intensity of 0.78 MW/cm^2^, demonstrating the favorable nonlinear optical properties of the Y_2_O_3_-based saturable absorber. Compared with previously published Y_2_O_3_ thin film based saturable absorbers, Y_2_O_3_ aerogel saturable absorbers enhance light–matter interactions due to their high porosity and large surface area, enabling a variety of mode-locking operations. These results show that amorphous silicon-doped Y_2_O_3_ aerogel is a highly promising optical material and will be extensively studied and applied in the field of ultrafast photonics in the near future. This reminds us that in future work, by doping different elements or doping different amounts of the same element into amorphous Y_2_O_3_ aerogel material, we can enhance its optical properties to make it more in line with people’s expectations.

## Supplementary Material

Supplementary Material Details
