# Supplementary material for: Preparation of amorphous silicon-doped Y2O3 aerogel enabling nonlinear optical features for ultrafast photonics
Source: Nanophotonics. 2024 Mar 13;13(9):1611–21. doi: 10.1515/nanoph-2023-0894 (PMC11636412; doi:10.1515/nanoph-2023-0894)
Supplement: Supplementary file 1 — Supplementary Material Details [file j_nanoph-2023-0894_suppl_001.docx]

Supplementary Material

**Preparation of amorphous silicon-doped Y_2_O_3_ aerogel enabling nonlinear optical features for ultrafast photonics**

Qingxi Zhao^1^ , Hongwei Chu*^,1^ , Zhongben Pan^1^ , Benxue Liu*^,2^ , Han Pan^1^, Shengzhi Zhao^1^ and Dechun Li*^,1^

*^1^ School of Information Science and Engineering, Key Laboratory of Laser and Infrared System of Ministry of Education, Shandong University, Qingdao 266237, China*

*^2^ Institute of Crystal Materials, State Key Laboratory of Crystal Materials, Shandong University, Jinan 250100, China*

*Corresponding Authors at:

*E-mail: [hongwei.chu@sdu.edu.cn](mailto:hongwei.chu@sdu.edu.cn). (Hongwei Chu)

*E-mail: [liubenxue@sdu.edu.cn](mailto:liubenxue@sdu.edu.cn). (Benxue Liu)

*E-mail: [dechun@sdu.edu.cn](mailto:dechun@sdu.edu.cn). (Dechun Li)

1. **Characterization of three Y_2_O_3_ aerogels**

Figures S1a and S1b are SEM images of the Y_2_O_3_-1 aerogel, and it can be seen that the Y_2_O_3_-1 aerogel is a porous nanomaterial. Figure S1c and S1d are TEM images of the Y_2_O_3_-1 aerogel. As can be seen from Figure S1d, blurred lattice streaks can be observed at high resolution, indicating that the Y_2_O_3_-1 aerogel is a poorly crystallized crystal. The element mapping images of Y_2_O_3_-1 aerogel are shown in Figure S1e and S1f, which clearly show the distribution of elements Y, and O.

**
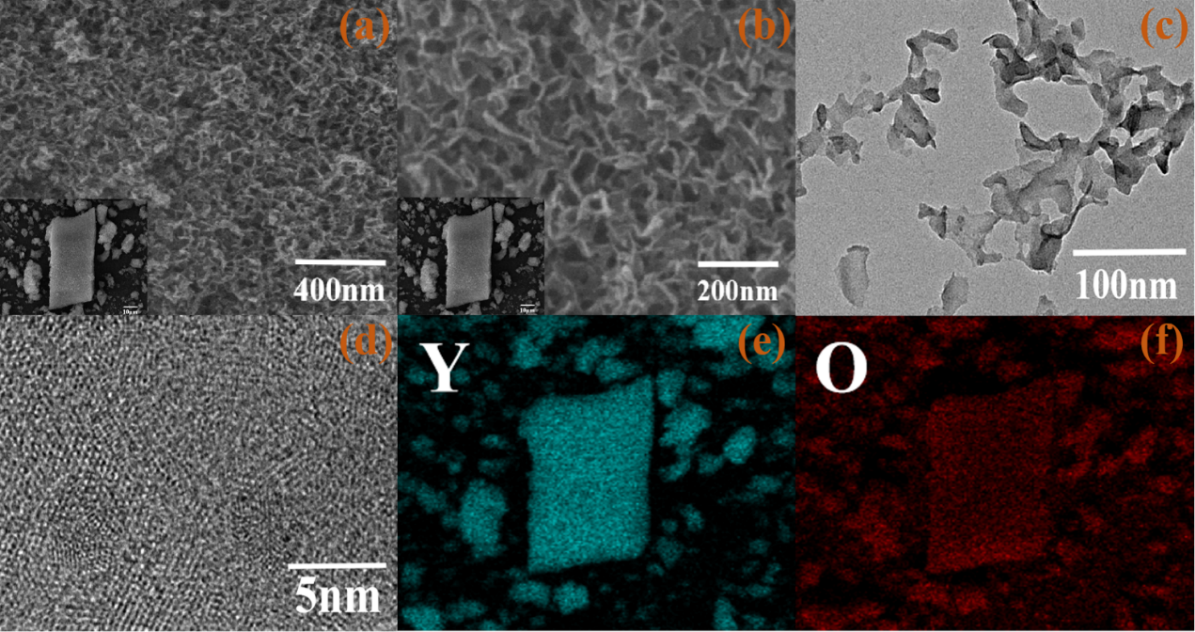
Figure S1**. Characterization of Y_2_O_3_-1 aerogel. (a) and (b) SEM images at different scales. (c) and (d) TEM images at different scales. (e) and (f) The energy-dispersive X-ray spectroscopy (EDS) elemental mappings of Y_2_O_3_-1 aerogel.

Figures S2a and S2b are SEM images of the Y_2_O_3_-2 aerogel, and it can be seen that the Y_2_O_3_-2 aerogel is also a nanoporous aerogel like Y_2_O_3_-3 aerogel. Figure S2c is TEM image of the Y_2_O_3_-2 aerogel. The corresponding element mapping images of Y_2_O_3_-2 aerogel are shown in Figures S2d, S2e and S2f, which clearly show the uniform distribution of elements Y, O and Si.

**
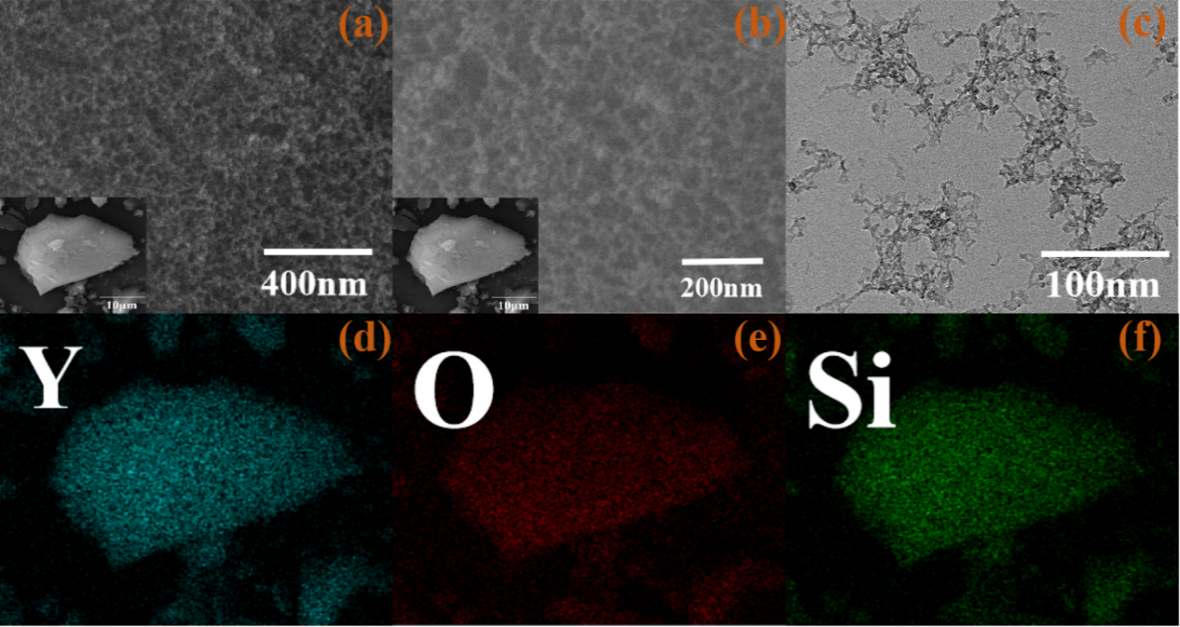
Figure S2**. Characterization of Y_2_O_3_-2 aerogel. (a) and (b) SEM images at different scales. (c) TEM image. (d), (e) and (f) The energy-dispersive X-ray spectroscopy (EDS) elemental mappings of Y_2_O_3_-2 aerogel.

In order to compare the effects of Si incorporation on the properties of Y_2_O_3_ aerogel, optical absorption characteristics and Fourier-transformed infrared (FT-IR) analysis were respectively characterized for the three aerogels, and the results were shown in Figure S3. As can be seen in Figure S3a, the optical absorption property of the three aerogels is similar. In Figure S3b, the wide peaks near 3450 cm^-1^ can be attributed to the stretching pattern of the hydroxyl group[1]. The peaks near 1550 cm^-1^ are due to C-O bond bending and stretching vibrations respectively[2], and these peaks are caused by the large specific surface area of the aerogels leading to the absorption of CO_2_ from the air. The peaks observed at 1400 cm^-1^ is attributed to the bending vibration mode of the water molecules [2]. The appearance of two peaks near 1000 cm^-1^ is due to the incorporation of Si resulting in the formation of Si-O bond. Finally, peaks obtained below 580 cm^-1^ are attributed to the metal oxide (Y-O) mode[3].

**
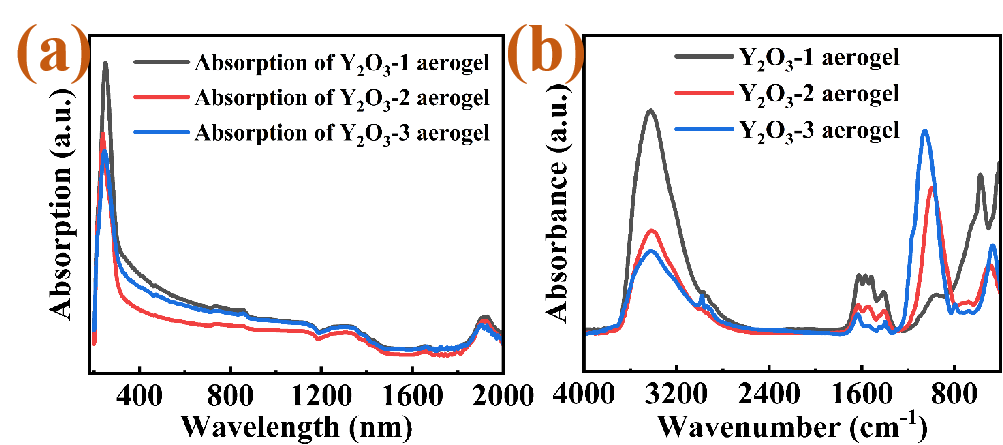
Figure S3**. Comparison of properties of three Y_2_O_3_ aerogels. (a) UV-VIS-IR absorption spectra. (b)Fourier-transformed infrared spectra.

1. **Mode-locking EDFL using Y_2_O_3_-1 aerogel**

When the pump power was adjusted to 275.76mW, we achieved the output of soliton molecules pulses by tuning the polarization state, as shown in Figure S4. The optical spectrum reveals the central wavelength of the soliton molecules is about 1557.8nm, and the spectrum exhibits modulation with the modulation period of 0.38nm, as shown in the inset of Figure S4a. There is an obvious continuous wave component in the spectrum, and the central wavelength is 1545.1 nm, as shown in Figure S4a. The pulse trains diagram shown by the oscilloscope in Figure S4b has an interval of 131.8ns between adjacent pulses, which matches the repetition frequency at this time. In Figure S4c, the SNR is about 50.5dB, and the repetition frequency is 7.587 MHz. We notice that the repetition frequency at this time (7.587 MHz) is different from the previous repetition frequency (7.487 MHz), which is caused by a slight change in the single-mode fiber length when the SA is replaced. The inset of Figure S4c shows the RF spectrum in the 1GHz range, which shows the stability of mode-locking state. The autocorrelation trace and multi-peak fitting curve are shown in Figure S4d, where the FWHM of the highest peak is 2.56 ps, corresponding to the pulse duration of 1.66 ps. The pulse interval is 21.23 ps, which matches well with the modulation period.


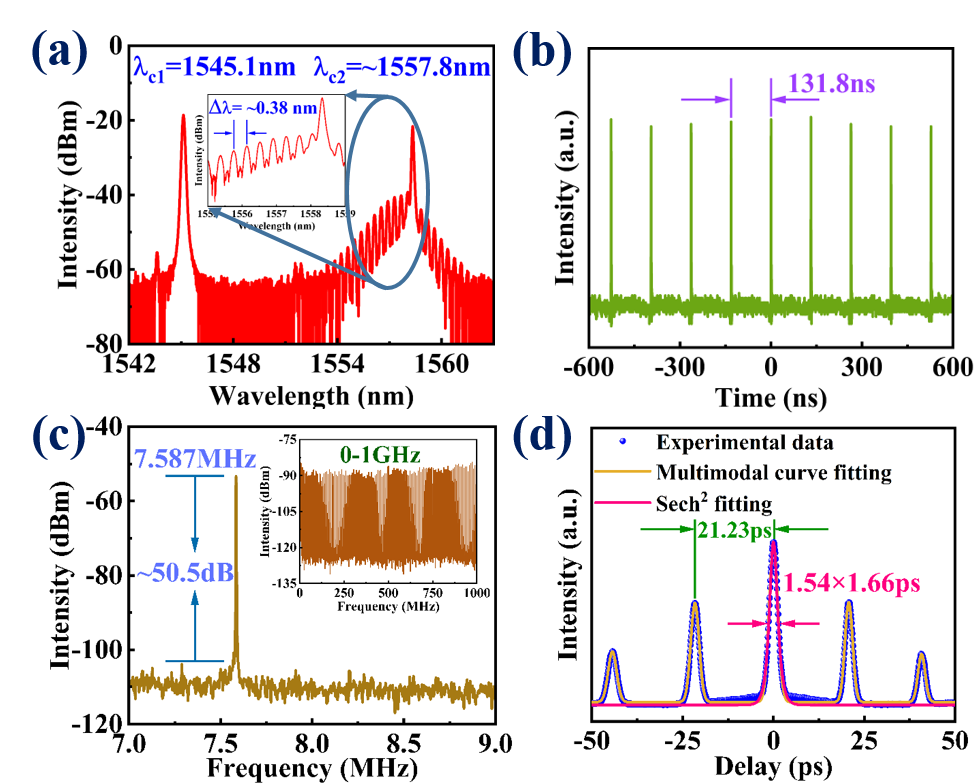


**Figure S4.** Output performance of the mode-locking EDFL using Y_2_O_3_-1 aerogel. (a) Optical spectrum. (b) Pulse trains. (c) RF spectra. (d) Autocorrelation trace.

1. **Mode-locking EDFL using Y_2_O_3_-2 aerogel**

By tuning the polarization state and pump power, the mode-locking pulses near 1550nm are realized, as shown in Figure S5 and Figure S6. Figure S5 shows that the laser is in the conventional soliton mode-locking operation when the pump power is 275.76 mW. The optical spectrum in Figure S5a shows a clear Kelly sideband with a central wavelength of 1555.7 nm and a 3 dB spectral bandwidth of 3.96nm. The pulse trains diagram in Figure S5b shows the time interval between adjacent pulses, which is 135.2ns. Due to the replacement of the SA, the cavity length of the resonator has changed a little. Figure S5c shows the RF spectra with the central frequency of 7.396MHz and the SNR of about 45.1dB. The stability of the mode-locking state was confirmed by the RF spectrum in the 1GHz range, as shown in the inset of Figure S5c. The autocorrelation trace in Figure S5d revealed the FWHM of 2.20 ps, with a corresponding pulse width of 1.43 ps after hyperbolic secant fitting.


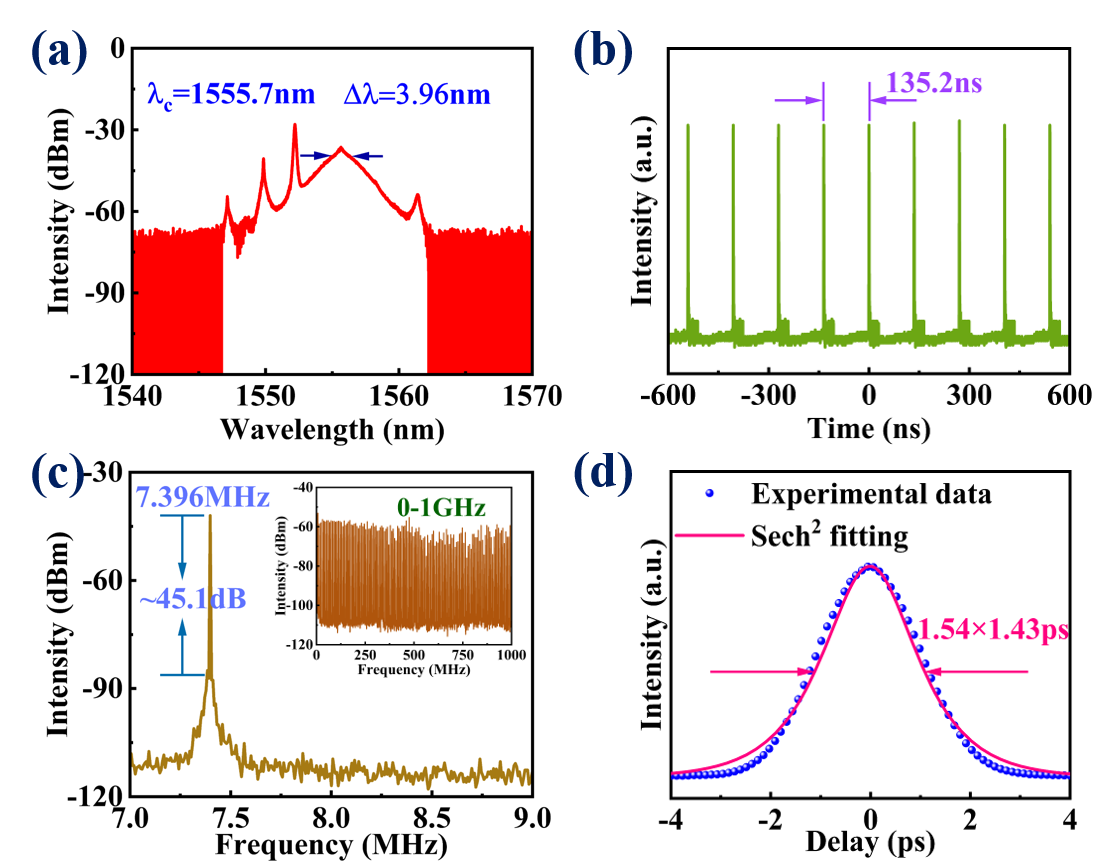


**Figure S5.** Output performance of the conventional soliton pulse mode-locking operation using Y_2_O_3_-2 aerogel. (a) Optical spectrum. (b) Pulse trains. (c) RF spectra. (d) Autocorrelation trace.

When the pump power is 275.76 mW, we get the output of the noise-like pulse mode-locking operation as shown in Figure S6 by adjusting the PC. The central wavelength of about 1554.3 nm and a 3dB spectral bandwidth of about 6.0nm were realized, as depicted in Figure S6a. The wide and smooth spectrum is typical characteristic of the noise-like pulses, but there is an obvious continuous wave component in the spectrum. The pulse trains diagram, as shown in Figure S6b, has a pulse interval of 135.2 ns. In Figure S6c, the repetition frequency is 7.396 MHz, which is consistent with the conventional soliton mode-locking operation, with an SNR of about 36.8 dB. The inset displays the RF spectrum within a range of 1 GHz. Figure S6d shows the autocorrelation trace and the fitting curve, respectively. From the inset, we can see that the pulse duration of the narrow coherent peak is 1.08 ps on the broad pedestal, which further proves that the output pulse is a noise-like pulse.


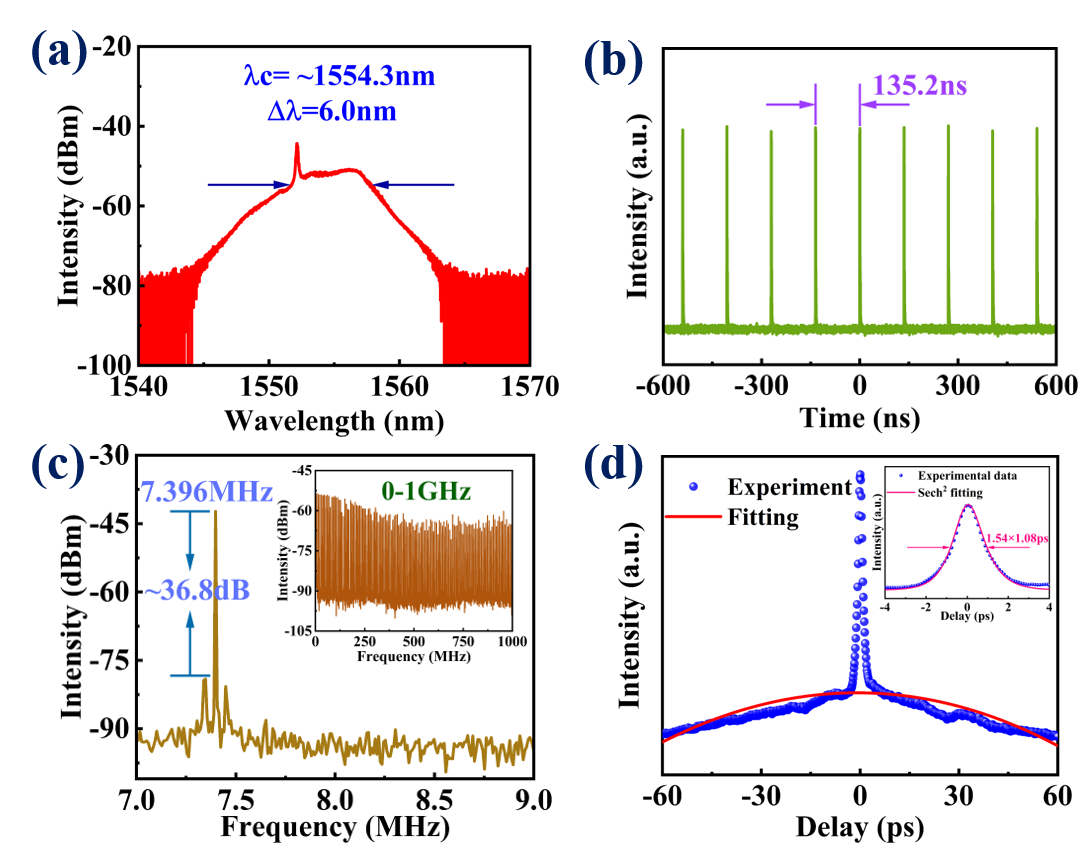


**Figure S6.** Output performance of the noise-like pulse mode-locking operation using Y_2_O_3_-2 aerogel. (a) Optical spectrum. (b) Pulse trains. (c) RF spectra. (d) Autocorrelation trace.

1. **Fabrication of Y_2_O_3_–based SA**

For Y_2_O_3_-1 aerogel and Y_2_O_3_-2 aerogel, the minimum waist diameter of the fabricated tapered fiber is 14.3 µm and 12 µm, respectively, and the length of the tapered area is about 5.4 mm and 5.5 mm, respectively.


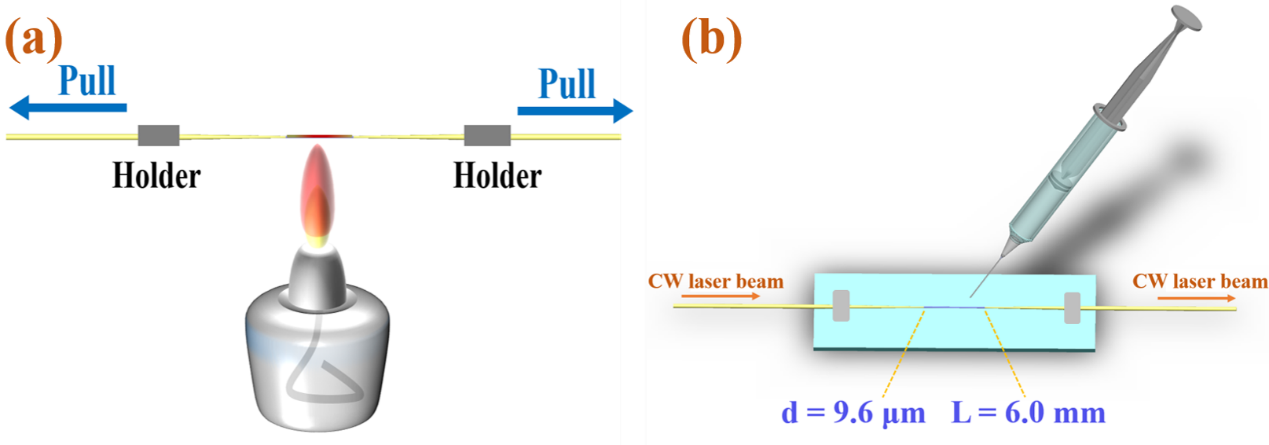


**Figure S7.** Fabrication of Y_2_O_3_–based SA. (a) Diagram of preparing tapered fiber. (b) Diagram of depositing material onto the tapered area.

1. **In-line twin-balanced-detector system**

**
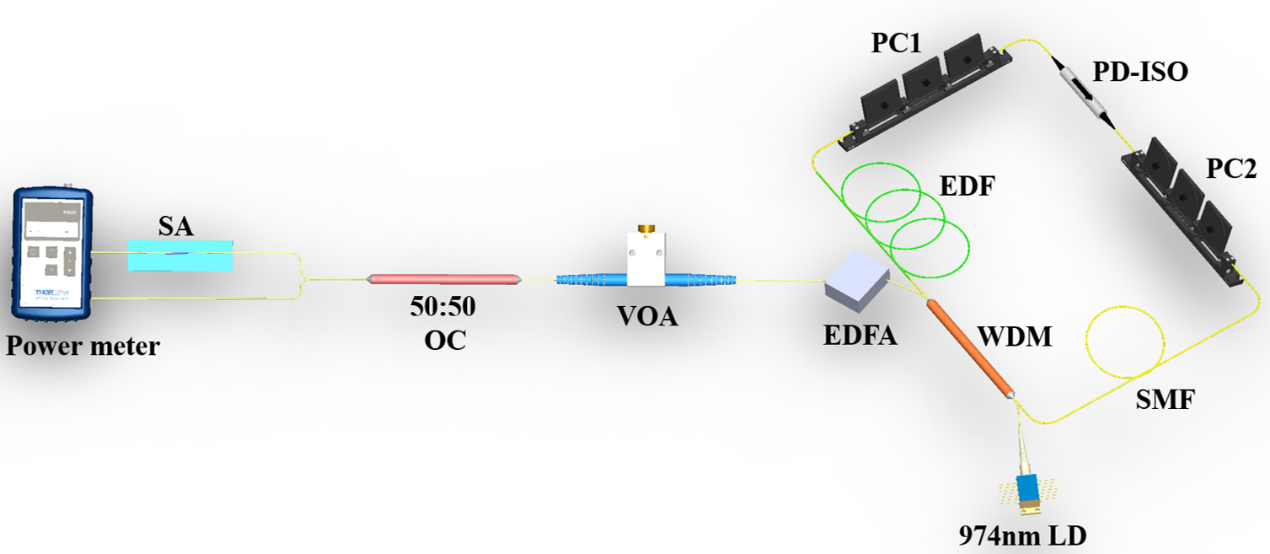
**

**Figure S8.** Schematic diagram of the in-line twin-balanced-detector system.

1. **Laser configuration**

**
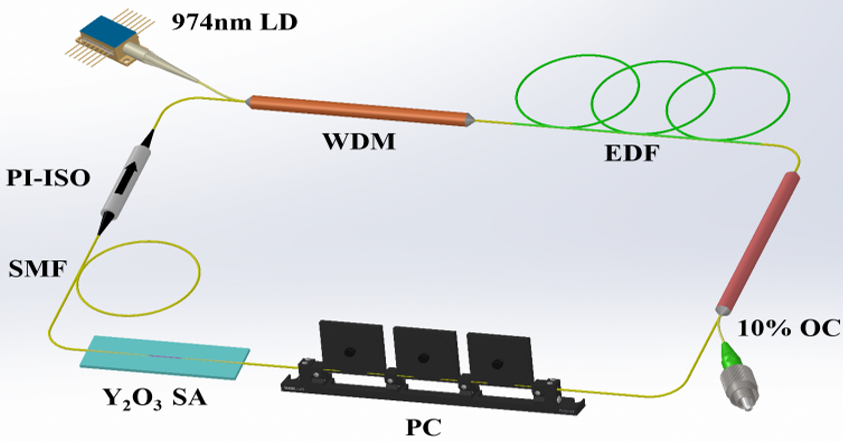
**

**Figure S9.** Set-up diagram of the EDFL.

1. **Related reaction equations in the formation of Y_2_O_3_ aerogel**


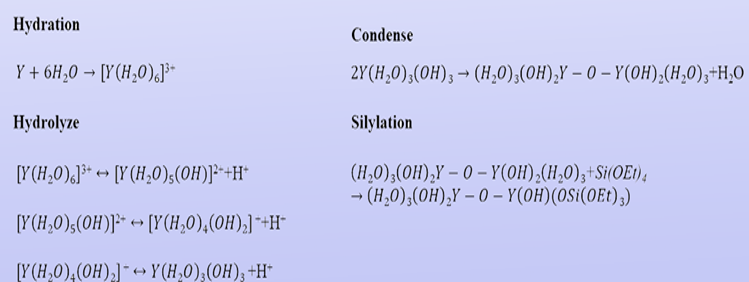
**Figure S10.** Related reaction equations in the formation of Y_2_O_3_ aerogel.

**References**

[1] P. Jeevanandam, Y. Koltypin, O. Palchik, and A. Gedanken, "Synthesis of morphologically controlled lanthanum carbonate particles using ultrasound irradiation," *Journal of Materials Chemistry,* vol. 11, no. 3, pp. 869-873, 2001.

[2] S. Som and S. Sharma, "Eu3+/Tb3+-codoped Y2O3 nanophosphors: Rietveld refinement, bandgap and photoluminescence optimization," *Journal of Physics D: Applied Physics,* vol. 45, no. 41, p. 415102, 2012.

[3] R. Mangalaraja, J. Mouzon, P. Hedström, C. P. Camurri, S. Ananthakumar, and M. Odén, "Microwave assisted combustion synthesis of nanocrystalline yttria and its powder characteristics," *Powder Technology,* vol. 191, no. 3, pp. 309-314, 2009.
